# Heart Rate Reduction and Outcomes in Heart Failure Outpatients

**DOI:** 10.3390/jcm12216779

**Published:** 2023-10-26

**Authors:** Felix Memenga, Meike Rybczynski, Christina Magnussen, Alina Goßling, Christoph Kondziella, Nina Becher, Peter Moritz Becher, Julia Bernadyn, Filip Berisha, Wiebke Bremer, Christoph Sinning, Stefan Blankenberg, Paulus Kirchhof, Dorit Knappe

**Affiliations:** 1 Department of Cardiology, University Heart & Vascular Center Hamburg, 20246 Hamburg, Germany; m.berner@uke.de (M.R.); c.magnussen@uke.de (C.M.); a.gossling@uke.de (A.G.); c.kondziella@uke.de (C.K.); n.fluschnik@uke.de (N.B.); m.becher@uke.de (P.M.B.); julia.bernadyn@gmx.de (J.B.); f.berisha@uke.de (F.B.); s.blankenberg@uke.de (S.B.); p.kirchhof@uke.de (P.K.); d.knappe@uke.de (D.K.); 2 German Center for Cardiovascular Research (DZHK), Partner Site Hamburg/Kiel/Luebeck; 3 Institute of Cardiovascular Sciences, University of Birmingham, Birmingham B152TT, UK

**Keywords:** heart failure, heart rate reduction, beta blocker, ivabradine

## Abstract

**Aim.** Pharmacologic reduction in heart rate with beta-blockers (BB) or ivabradine is associated with improved survival in heart failure (HF) with sinus rhythm. We analyzed the association of different heart rate-reducing drug treatments on outcomes in HF outpatients. **Methods.** Consecutive patients with HF in sinus rhythm referred to a specialized tertiary service were prospectively enrolled from August 2015 until March 2018. Clinical characteristics were assessed at baseline. We performed Cox regression analyses to examine the effect of the resting heart rate and different heart rate-reducing drug regimens on all-cause mortality and a composite endpoint of “all-cause mortality or heart transplantation” over a mean follow-up of 3.1 years. **Results.** Of the 278 patients included, 213 (76.6%) were male, the median age was 57.0 years (IQR 49.0–66.1), and 185 (73.7%) had an ejection fraction <40%. Most patients received BB in submaximal [n = 118] or maximum dose [n = 136]. Patients on BB in maximum dose plus ivabradine [n = 24] were younger (53.0 vs. 58.0 years) and had a lower EF (25 vs. 31%). Higher resting heart rate was associated with an increased risk of death or transplantation (HR 1.03 [1.01, 1.06], p = 0.0072), even after adjusting for age and sex. There were no differences between the groups concerning all-cause mortality or the composite endpoint. **Conclusion.** Our prospective study confirms the association between low heart rate and survival in HF patients receiving various heart rate-reducing medications. We could not identify a specific effect of either regimen.

## 1. Introduction

Epidemiological studies demonstrate a correlation between resting heart rate (HR) and cardiovascular morbidity, and the beneficial effects of heart rate reduction in heart failure (HF) are well established [1,2,3,4]. In HF with reduced ejection fraction (HFrEF), beta-blockers (BB) improve survival, hospitalization rates, and cardiac function via reducing sympathetic activity, HR, and myocardial oxygen consumption, and have been a major pillar in the pharmacological treatment of HFrEF for more than two decades [2,5,6]. 

In the 2010 SHIFT study, the selective sinus node (I_F_) inhibitor ivabradine proved similarly beneficial in HF patients with an EF of 35% or lower and (despite not reducing all-cause and cardiovascular mortality) underlined the important role of the HR in HF pathophysiology [7]. 

The 2021 ESC guidelines for the diagnosis and treatment of acute and chronic heart failure recommend ivabradine in symptomatic HF patients with an EF of ≤35% in sinus rhythm (SR) and a resting HR ≥ 70 bpm, despite treatment with evidence-based BB (or a maximum tolerated dose below that), or in patients who are unable to tolerate or have contraindications for a BB [8]. While BB in HFrEF have a class I recommendation, ivabradine is issued a class IIa recommendation and should be used in combination with an ARNI (angiotensin receptor neprilysin inhibitor) or ACEI/ARB (angiotensin-converting-enzyme inhibitor/angiotensin receptor blocker) and MRA (mineralocorticoid receptor antagonist) [8]. 

While only 26% of subjects in the SHIFT study were on target doses of BB and only 56% on at least half-target doses of BB [7], concerns have been raised that, due to BB underuse and underdosing, the benefits of ivabradine in SHIFT may have been overestimated. Furthermore, the effects of ivabradine in the group of patients on more than 50% of BB target dose are attenuated [7]. Whether there is a true “synergistic” effect of ivabradine in addition to BB treatment remains elusive, as the benefits of ivabradine may rather be limited to patients with (partial or complete) BB intolerance, when ivabradine is used as “replacement BB”. 

To further elucidate the value of HR-lowering therapies in the form of ivabradine in combination with BB therapy and the impact of BB dosing on outcomes in HF, we conducted this prospective study.

## 2. Methods

### 2.1. Study Population

The study population consisted of a prospectively enrolled “real-world” cohort of outpatients with HF referred for further evaluation of HF treatment, such as LVAD (left ventricular assist device) and heart transplantation, to our specialist HF service. All patients referred for specialist treatment of either known or recently diagnosed advanced HF between August 2015 and March 2018, who were in sinus rhythm at the time point of screening, were considered eligible to participate. Patients aged <18 years, with complete or partial BB intolerance, treatment with antiarrhythmic drugs (amiodarone or sotalol) or cardiac glycosides, and heart transplant recipients were excluded from the study.

Clinical variables included age, sex, weight, height, body mass index (BMI), systolic and diastolic blood pressure, and type of cardiomyopathy, as well as New York Heart Association (NYHA) class and 6 min walk distance as functional parameters, were assessed at baseline. Cardiac history and non-cardiac comorbidities (namely arterial hypertension, hypercholesterolemia, diabetes, chronic obstructive pulmonary disease (COPD), asthma or other lung diseases, chronic renal failure, history of severe hepatic failure, transient ischemic attack/ischemic stroke in history, hemorrhagic stroke, peripheral artery disease, and hyper- and hypothyroidism) were physician-diagnosed. All patients underwent standardized imaging by echocardiography. Echocardiographic measurements, including systolic (left-ventricular ejection fraction (LVEF) with the biplane Simpson’s method) and diastolic function (using doppler patterns of mitral valve inflow and tissue doppler), HF medication, current device therapy such as pacemaker, implantable cardioverter defibrillator (ICD) or cardiac resynchronization therapy (CRT), and laboratory measurements, were assessed at baseline as well. In terms of BB doses, a daily intake of 10 mg bisoprolol, 190 mg metoprolol succinate or 200 mg metoprolol tartrate, and 25 mg or 50 mg carvedilol (depending on body weight) or 10 mg nebivolol was considered as “maximum dose”.

The follow-up was obtained by regular clinical review. Information on outpatient and inpatient visits was captured electronically. All-cause death data were obtained from the death register. The study was approved by the local ethics committee (PV 6079) and conducted in concordance with the Declaration of Helsinki. Written informed consent was obtained from all participants.

### 2.2. Statistical Analysis

Continuous variables were presented as median (25th percentile, 75th percentile) and binary variables as absolute numbers (relative frequencies). For between-group comparisons, the Kruskal–Wallis test was used for continuous variables and the χ^2^ test for binary variables. 

The median follow-up time was estimated by the Kaplan–Meier potential follow-up estimator [9].

The outcome parameters of the analysis were ‘death from any cause’ and a composite endpoint of ‘death from any cause or heart transplantation’ (representing disease progression) during follow-up. The hazard ratios of the resting heart rate and different HR-reducing drug/dosing regimens were calculated through several univariable Cox regression models. We designed Kaplan–Meier curves for all-cause mortality and the composite endpoint. Survival curve differences were compared using the log-rank test.

A two-tailed *p*-value < 0.05 was considered statistically significant. All calculations were performed using R Version 4.0.3 [10].

## 3. Results

**Figure 1:**
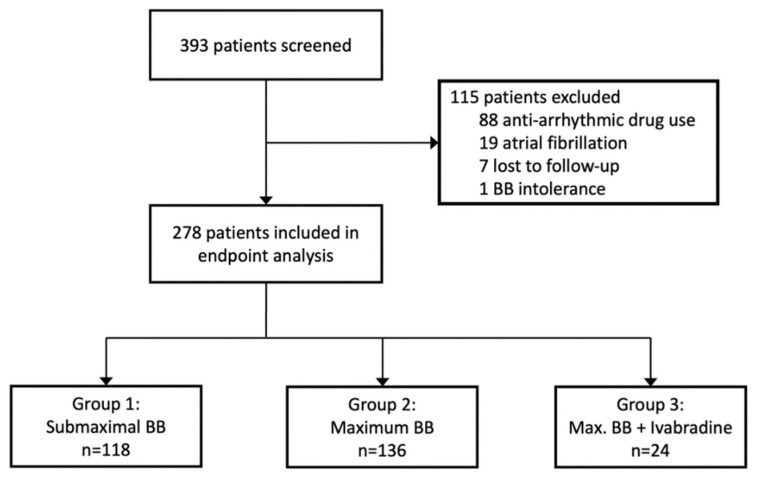
Profile of study screening and study groups.

Of the 393 patients screened, 88 patients were excluded due to intake of antiarrhythmic drugs or treatment with cardiac glycosides, 19 due to atrial fibrillation in the screening ECG, 7 were lost to follow-up, and 1 was excluded for complete BB intolerance (Figure 1). Thus, 278 patients were included in the analysis, of whom 213 (76.6%) were male. The median age was 57.0 years (interquartile range 49.0–66.1). HF was predominantly due to non-ischemic compared to ischemic origin (59.7% vs. 40.3%). Almost three-quarters of patients (73.7%) had HFrEF (LVEF < 40%), one quarter (25.1%) had HF with mildly reduced ejection fraction (HFmrEF, EF between 40% and 50%), and the majority had symptoms rated according to the New York Heart Association (NYHA) as class I-II/II (44.7%) or II-III/III (30.1%).

Most patients were treated with BB in submaximal [n = 118, 42%, *group 1*] or maximum dose [n = 136, 49%, *group 2*] without ivabradine (Figure 1). The BB most used were bisoprolol (n = 109), followed by metoprolol (n = 93), carvedilol (n = 65), and nebivolol (n = 11). Concerning metoprolol, 89 patients received metoprolol succinate and 4 patients metoprolol tartrate. Patients receiving ivabradine on top of BB in maximum dose [n = 24, 9%, *group 3 or “ivabradine group”*] were of younger age (53.0 vs. 58.0 years), had a lower LVEF (25 vs. 31%), and higher NT-proBNP levels (2089 [686, 3401] vs. 1008 [401, 2616]) than those only on BB treatment. HF etiology and most of the cardiac and non-cardiac comorbidities were evenly distributed between the study groups (Table 1). Use of other current guideline-recommended HF medication was decent throughout all participants (with an overall MRA use of 82% and an overall RAS blocker [ARNI/ACEI/ARB] use of 97%) and did not differ between the study groups. There was a higher use of cardiac resynchronization therapy (CRT) among those receiving ivabradine on top of BB (group 3, Table 2). Resting HR at baseline was 66 (group 1), 68 (group 2), and 76 bpm (group 3), respectively. Regarding the type of underlying cardiomyopathy, the majority of the patients presented with either dilative cardiomyopathy or ischemic cardiomyopathy (both 39%); the cohort with toxic cardiomyopathy included 5 patients associated with oncologic treatment and 2 patients with alcohol abuse. The cohort with other types of cardiomyopathy included, in the majority of patients, myocarditis, peripartum cardiomyopathy, and sarcoidosis, leaving a few unclassified patients.

**Figure 2:**
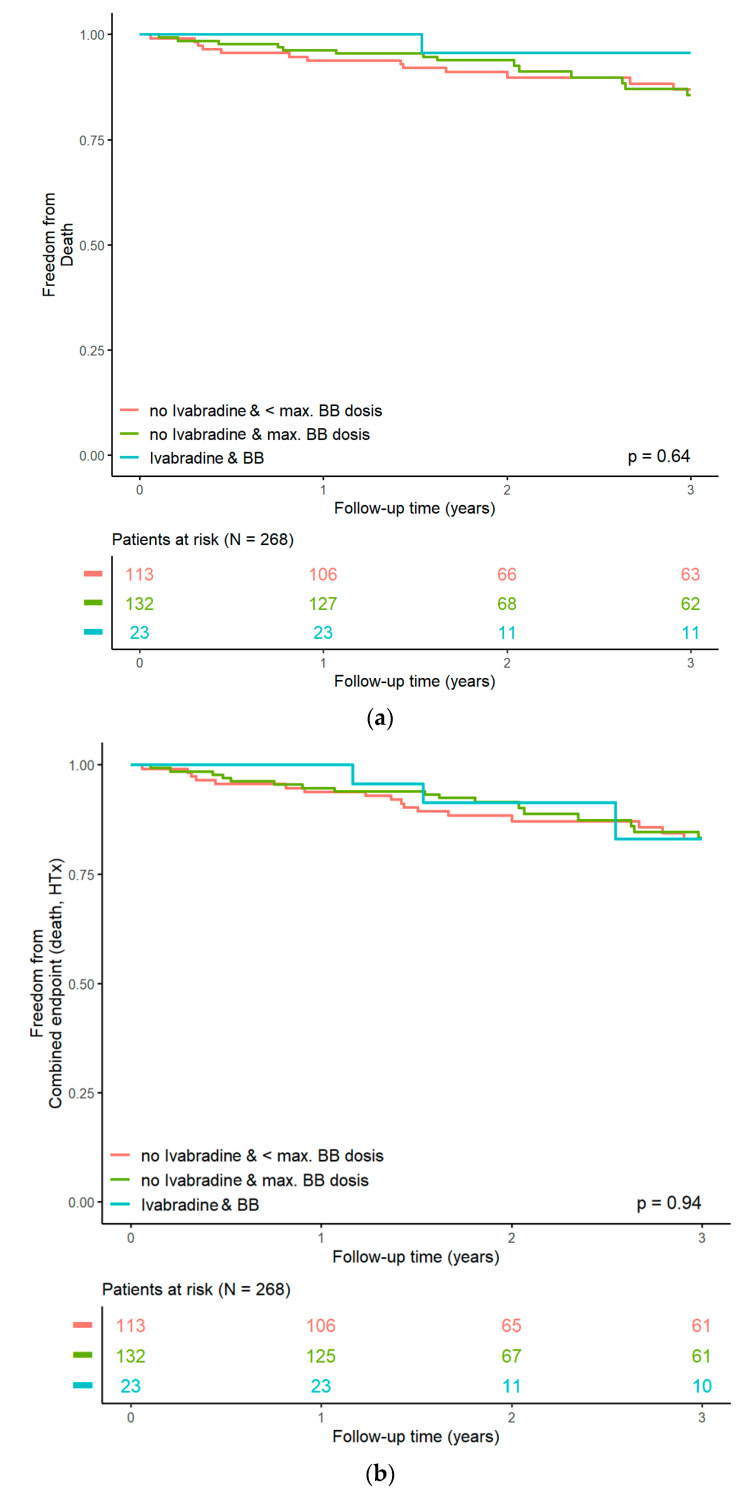
(**a**): Kaplan–Meier curves for all-cause mortality. (**b**): Kaplan–Meier curves for the composite endpoint (“death of any cause and heart transplantation”).

During a median follow-up of 3.14 (3.12–3.36) years, 30 deaths occurred, and 11 patients underwent heart transplantation, adding up to 40 composite outcome events (avoiding double-counting), which corresponds to an event rate of 19.2% over the total follow-up. There were 12 deaths in the first year, 7 deaths in the second year, and 9 deaths in the third year. Upon regression analysis, resting HR at the screening was associated with an increased risk of death or heart transplantation (hazard ratio HR 1.03 [1.01, 1.06], *p* = 0.0072). This effect persisted in a Cox regression model adjusting for age and sex (HR 1.03 [1.01, 1.06], *p* = 0.0061]. Compared to group 1 (BB in submaximal dose), we were not able to demonstrate a significantly reduced risk of “death of any cause” or “death of any cause or heart transplantation” in the group of patients with BB in maximum dose (group 2, HR 0.93 [0.49, 1.77], *p* = 0.83) or ivabradine “on top” (group 3, HR 0.84 [0.25–2.87], *p* = 0.79, Figure 2a,b), even after adjusting for age and sex. 

Also, both the BB dose (HR 1.0 [0.54, 1.86], *p* = 1.0) and the ivabradine dose (HR 1.13 [0.77, 1.66], *p* = 0.54) did not affect outcomes.

## 4. Discussion

In this prospective ‘all-comers’ cohort of patients referred to a specialized tertiary HF outpatient service, resting HR was associated with an increased risk of death or heart transplantation. In a comparison of groups of patients on different doses or regimes of HR-lowering pharmacotherapies, but with comparable baseline characteristics and use of other HF medication, we found a similar risk of adverse HF outcomes. Our study thus failed to demonstrate benefits of BB up-titration or the addition of ivabradine to BB therapy on all-cause mortality or a disease-related combined endpoint.

Drawing prospective patients from a large tertiary HF center, this analysis confirms the well-established and recognized adverse effect of an elevated HR on prognosis in HF [4,11], which was underpinned by the SHIFT study of 2010 [7]. However, while SHIFT demonstrated an improvement in outcomes through HR reduction with ivabradine in an ambulatory HFrEF (LVEF ≤ 35%) population with SR (≥70 bpm), the benefits of HR reduction in more advanced stages of HF are less well studied, even though a post hoc analysis of SHIFT suggested beneficial effects of ivabradine on HF outcomes independent of HF severity [12]. 

Our study cohort is comprised of ambulatory HF patients referred for advanced HF treatment options. It is characterized by a decent and widespread use of guideline-recommended HF medication including RAS-Blockers and MRA, as well as device therapy, throughout all study participants, which was a prerequisite for comparing different HR-reducing drug regimens. However, despite the good and homogeneous HF pharmacotherapy in our study and the relatively young age, the mortality rate in our study is high compared to other chronic HF studies and registries [13,14]. This may be due to a more advanced nature of ambulatory HF, owing to the setting in specialized outpatient care, which may especially apply to the “ivabradine group” with a worse LVEF and higher NT-proBNP, despite the younger age. Representing disease progression without further management options both drug-wise and device-wise, we therefore chose a composite “disease-related” endpoint of “heart transplantation or death of any cause”.

In addition to the lower mean LVEF, patients on ivabradine had a higher mean resting HR (76 bpm) than those on BB “monotherapy”, which not only confirms the guideline-coherent use of ivabradine in our study but could moreover be regarded as a hint at its beneficial effects in advanced HF. Despite “negatively” predisposing characteristics at baseline—both an elevated HR and a lower LVEF inversely affect prognosis in HF [4,11,15,16]—there was no higher risk of adverse outcomes. Even though there was a higher use of CRT in the “ivabradine group”, which may affect outcomes, our data thus support the use of ivabradine in further advanced HF stages.

Finally, there is, so far, conflicting evidence regarding the up-titration of BB but also RAS blockers and MRAs in HF. Biomarker-based up-titration schemes may have a superior effect on HF outcomes compared to up-titration to guideline-recommended doses, which are often not reached [17]. BB up-titration may not even improve outcomes in older patients [18]. In clinical practice, the timely implementation of all evidence-based drug classes (currently BB, SGLT2 inhibitors, ARNIs, and MRA) and up-titration to target doses afterwards has been proposed as the preferred sequencing strategy in ambulatory HFrEF patients [19]. 

## 5. Limitations

As this analysis drew “real-world” data from a large tertiary heart failure center resulting in good and homogeneous therapy, treatment groups were not randomized. Also, the small sample size, which allowed for a detailed characterization of patients, is—along with the single-center nature of the study—a major limitation that reduces statistical power and limits generalizability. The small number of events, however, mirrors the extent of risk reduction achievable through the consequent use of guideline-recommended prognosis-modifying treatments, both pharmacologically and device-driven. 

These pharmacological prognosis-modifying treatments, however, included neither SGLT2 inhibitors (which were lacking FDA or EMA approval for HF treatment by the end of study recruitment in March 2018) nor cardiac glycosides (which were an exclusion criterion to our study for also lowering HR), both currently recommended in ESC HF guidelines (class of recommendation I and II, respectively) [8]. In addition, owing to the “real-world” setting, patients on metoprolol tartrate were not switched to metoprolol succinate, which is the approved molecule in HFrEF therapy [20]. 

In addition, there were no follow-up assessments of heart rate changes during the follow-up.

Moreover, we saw a gender imbalance, with a predominance of male study participants (~77%), which has been observed in many recent HF trials and registries [21,22] and may be due to complex and multi-dimensional reasons. Even though we used Cox regression models, adjusting for age and sex, the study’s findings should therefore primarily be regarded as hypothesis-generating, but validation in larger HF cohorts on contemporary guideline-recommended therapies can mitigate these limitations.

## 6. Conclusions

Our prospective study underlines the importance of heart rate reduction in HF with SR but failed to demonstrate a better risk reduction in the group of patients with up-titrated BB doses or on a combination of BB plus ivabradine. Despite unfavorable clinical characteristics at baseline, patients receiving a combination of ivabradine and BB, on the other hand, did not have a higher risk of adverse outcomes.

## Figures and Tables

**Table 1:** Baseline characteristics stratified by the study groups.

		1	2	3	
	Total	BB in Submax. Dose	BB in Max. Dose	BB in Max. Dose + Ivabradine	*p*-Value
*N*	278	118	136	24	
**Clinical variables**					
Male gender (%)	213 (76.6)	83 (70.3)	112 (82.4)	18 (75.0)	0.077
Age (years)	57.0 (49.0, 66.1)	60.0 (51.0, 67.1)	56.0 (48.0, 66.0)	53.0 (43.7, 58.2)	0.031
Height (cm)	177 (170, 184)	174 (168, 180)	180 (172, 186)	178 (171, 183)	0.0014
weight (kg)	83.0 (72.0, 96.3)	76.0 (67.8, 88.1)	88.0 (74.3, 100.8)	87.0 (77.2, 99.8)	<0.001
BMI (kg/m^2^)	26.0 (23.2, 29.9)	25.4 (22.9, 28.6)	27.0 (24.1, 31.0)	28.7 (24.7, 33.5)	0.0089
Systolic blood pressure (mmHg)	115 (100, 130)	114 (100, 130)	116 (105, 135)	112 (95, 118)	0.17
Diastolic blood pressure (mmHg)	69 (60, 78)	70 (59, 77)	67 (60, 78)	69 (60, 80)	0.89
**Type of cardiomyopathy, n (%)**					
Dilated cardiomyopathy	108 (38.99)	35 (29.66)	61 (45.19)	12 (50.00)	0.021
on LVAD support	1 (0.36)	0 (0)	1 (0.74)	0 (0)	0.59
Ischemic cardiomyopathy	108 (38.99)	43 (36.44)	53 (39.26)	12 (50.00)	0.46
on LVAD support	3 (1.08)	3 (2.54)	0 (0)	0 (0)	0.13
Ischemic/dilated cardiomyopathy	1 (0.36)	0 (0)	1 (0.74)	0 (0)	0.59
Hypertrophic cardiomyopathy	1 (0.36)	0 (0)	1 (0.74)	0 (0)	0.59
Hypertrophic obstructive cardiomyopathy	1 (0.36)	1 (0.85)	0 (0)	0 (0)	0.51
Restrictive cardiomyopathy	5 (1.81)	3 (2.54)	2 (1.48)	0 (0)	0.64
Valvular cardiomyopathy	7 (2.53)	5 (4.24)	2 (1.48)	0 (0)	0.27
Toxic cardiomyopathy	7 (2.53)	6 (5.08)	1 (0.74)	0 (0)	0.064
Non-compaction cardiomyopathy	1 (0.36)	0 (0)	1 (0.74)	0 (0)	0.59
Others	33 (11.91)	21 (17.80)	12 (8.89)	0 (0)	0.016
**Functional parameters**					
NYHA class: I (%)	59 (23.32)	31 (28.70)	21 (17.07)	7 (31.82)	0.070
NYHA class: II (%)	107 (42.29)	41 (37.96)	56 (45.53)	10 (45.45)	0.48
NYHA class: III (%)	58 (22.92)	26 (24.07)	29 (23.58)	3 (13.64)	0.55
NYHA class: IV (%)	1 (0.40)	1 (0.93)	0 (0)	0 (0)	0.51
NYHA class: I–II (%)	6 (2.37)	3 (2.78)	3 (2.44)	0 (0)	0.74
NYHA class: II–III (%)	20 (7.91)	5 (4.63)	13 (10.57)	2 (9.09)	0.24
NYHA class: III–IV (%)	2 (0.79)	1 (0.93)	1 (0.81)	0 (0)	0.90
6 min walk test (m)	370.84 ± 145.25	340.43 ± 147.15	376.14 ± 149.16	427.67 ± 134.46	0.47
**History of comorbidities, n (%)**					
Arterial hypertension	137 (50.55)	60 (52.17)	65 (48.87)	12 (52.17)	0.86
Hypercholesterolemia	98 (42.42)	38 (39.18)	50 (44.25)	10 (47.62)	0.67
Diabetes	52 (19.05)	17 (14.91)	29 (21.32)	6 (26.09)	0.29
COPD	22 (9.48)	13 (13.54)	6 (5.26)	3 (13.64)	0.098
Asthma bronchiale	22 (9.48)	7 (7.29)	9 (7.89)	6 (27.27)	0.011
Other lung disease	25 (10.82)	9 (9.28)	13 (11.50)	3 (14.29)	0.76
Chronic renal failure	86 (37.23)	32 (33.68)	44 (38.60)	10 (45.45)	0.54
Severe hepatic failure	6 (2.60)	1 (1.04)	3 (2.65)	2 (9.09)	0.10
Transient ischemic attack/ Ischemic stroke	20 (8.66)	4 (4.08)	14 (12.50)	2 (9.52)	0.095
Hemorrhagic stroke	1 (0.44)	0 (0)	1 (0.91)	0 (0)	0.58
Peripheral arterial disease	9 (3.88)	4 (4.12)	5 (4.39)	0 (0)	0.62
Hyperthyroidism	19 (8.15)	8 (8.16)	11 (9.65)	0 (0)	0.33
Hypothyroidism	27 (11.64)	11 (11.34)	14 (12.28)	2 (9.52)	0.93
**Cardiac history, n (%)**					
Myocardial infarction	90 (38.79)	36 (37.11)	44 (38.94)	10 (45.45)	0.77
Cardiogenic shock	32 (15.38)	12 (13.79)	16 (15.69)	4 (21.05)	0.72
Left ventricular thrombus	26 (11.16)	8 (8.16)	14 (12.39)	4 (18.18)	0.34
Atrial fibrillation	57 (24.68)	23 (23.71)	32 (28.32)	2 (9.52)	0.18
Atrial flutter	12 (5.22)	8 (8.25)	4 (3.57)	0 (0)	0.17
Ventricular tachycardia	28 (12.44)	6 (6.32)	21 (19.09)	1 (5.00)	0.013
Ventricular fibrillation	16 (6.93)	2 (2.04)	10 (8.93)	4 (19.05)	0.011
**Electrocardiogram**					
Heart rate (bpm)	68.00 (60.00, 77.00)	66.00 (59.00, 77.83)	68.00 (60.00, 76.00)	75.50 (67.83, 81.00)	0.030
Atrial fibrillation (%)	0 (0)	0 (0)	0 (0)	0 (0)	
**Echocardiography**					
EF (Simpson) (%)	31.00 (25.00, 40.00)	35.00 (27.00, 42.00)	30.00 (25.00, 36.00)	25.00 (21.42, 31.17)	<0.001
EF <40% (%)	185 (73.71)	66 (63.46)	99 (80.49)	20 (83.33)	0.0078
EF 40–50% (%)	63 (25.10)	35 (33.65)	24 (19.51)	4 (16.67)	0.030
EF >50% (%)	3 (1.20)	3 (2.88)	0 (0)	0 (0)	0.12
Diastolic dysfunction: none (%)	45 (20.36)	21 (22.58)	22 (20.37)	2 (10.00)	0.45
Diastolic dysfunction I° (%)	98 (44.34)	40 (43.01)	48 (44.44)	10 (50.00)	0.85
Diastolic dysfunction II° (%)	48 (21.72)	20 (21.51)	24 (22.22)	4 (20.00)	0.97
Diastolic dysfunction: III° (%)	30 (13.57)	12 (12.90)	14 (12.96)	4 (20.00)	0.68
E/E’	11.25 (8.47, 15.39)	11.39 (8.17, 17.02)	10.20 (7.85, 15.08)	13.25 (11.11, 14.64)	0.10
E/A	1.14 (0.71, 1.94)	1.00 (0.70, 1.86)	1.19 (0.77, 1.83)	1.07 (0.69, 2.51)	0.77
RVP (mmHg)	29.00 (22.00, 37.00)	29.00 (21.20, 39.00)	28.00 (22.00, 34.83)	30.00 (25.00, 34.67)	0.64
TAPSE (mm)	18.60 (15.00, 21.39)	18.00 (14.07, 22.00)	19.00 (16.00, 21.00)	17.00 (13.17, 22.00)	0.79
Aortic valve stenosis moderate/severe (%)	2 (0.72)	1 (0.85)	1 (0.74)	0 (0)	0.90
Aortic valve regurgitation moderate/severe (%)	3 (1.08)	2 (1.69)	1 (0.74)	0 (0)	0.66
Mitral valve stenosis moderate/ severe (%)	1 (0.36)	0 (0)	1 (0.74)	0 (0)	0.59
Mitral valve regurgitation moderate/severe (%)	55 (19.78)	20 (16.95)	29 (21.32)	6 (25.00)	0.55
Tricuspid valve regurgitation moderate/severe (%)	34 (12.23)	10 (8.47)	18 (13.24)	6 (25.00)	0.070
Left atrial volume (mL)	69.03 (50.88, 91.13)	67.15 (44.33, 85.08)	70.10 (54.12, 95.30)	78.42 (46.94, 97.97)	0.22
Right atrial area (cm^2^)	16.55 (12.94, 20.50)	16.00 (12.38, 19.82)	16.80 (13.31, 20.64)	16.22 (13.55, 20.63)	0.29
IVSD (mm)	10.00 (8.00, 11.57)	10.00 (8.63, 12.00)	10.00 (8.00, 11.27)	9.90 (7.92, 11.00)	0.57
LVEDD (mm)	62.00 (56.00, 68.53)	58.35 (53.18, 64.91)	64.00 (59.00, 73.00)	66.00 (60.75, 70.08)	<0.001
**Laboratory data**					
Hemoglobin (g/dL)	13.70 (12.40, 14.60)	13.30 (12.00, 14.28)	13.90 (12.50, 14.70)	14.60 (13.13, 15.00)	0.0049
Ferritin (ug/L)	114.00 (64.00, 189.83)	104.00 (64.00, 175.00)	133.00 (59.83, 237.33)	91.00 (63.00, 170.08)	0.31
Transferrin (g/L)	2.60 (2.40, 3.00)	2.60 (2.40, 3.00)	2.60 (2.40, 2.90)	2.85 (2.60, 3.20)	0.034
Transferrin saturation (%)	24.50 (18.92, 30.00)	24.00 (18.42, 29.00)	26.00 (18.42, 31.00)	22.50 (19.83, 28.00)	0.10
Creatinine (mg/dL)	1.20 (0.97, 1.40)	1.11 (0.95, 1.40)	1.20 (1.07, 1.50)	1.10 (0.97, 1.37)	0.12
eGFR (mL/min/1.73 m^2^)	69.48 (51.60, 90.28)	70.19 (51.50, 90.21)	69.47 (49.46, 89.47)	69.48 (60.51, 91.97)	0.79
Creatin kinase (U/L)	105.00 (70.17, 147.00)	92.00 (67.00, 137.33)	113.00 (73.00, 157.67)	106.00 (86.33, 156.00)	0.20
NT-proBNP (ng/L)	1073.00 (407.33, 2704.33)	926.00 (334.83, 2417.25)	1109.50 (463.42, 2727.58)	2089.00 (685.83, 3401.33)	0.13

**Table 2:** HF therapies stratified by the study groups.

		1	2	3	
	Total	BB in Submax. Dose	BB in Max. Dose	BB in Max. Dose + Ivabradin	*p*-Value
*N*	278	118	136	24	
**HF medication, n (%)**					
Beta Blocker (%)	278 (100)	118 (100)	136 (100)	24 (100)	
MRA (%)	184 (81.78)	70 (75.27)	93 (84.55)	21 (95.45)	0.051
RAAS (%)	219 (96.90)	88 (94.62)	109 (98.20)	22 (100)	0.23
ACE-I (%)	124 (54.87)	56 (59.57)	62 (56.36)	6 (27.27)	0.021
ARB (%)	50 (22.03)	18 (19.15)	25 (22.52)	7 (31.82)	0.43
ARNI (%)	50 (22.22)	17 (18.48)	24 (21.62)	9 (40.91)	0.074
**Prior interventions, n (%)**					
Coronary stenting	76 (33.48)	34 (35.79)	33 (29.73)	9 (42.86)	0.42
Coronary artery bypass graft	26 (11.21)	12 (12.24)	13 (11.50)	1 (4.76)	0.61
Prior valve surgery *	33 (14.22)	15 (15.46)	18 (15.79)	0 (0)	0.15
MitraClip™ procedure	8 (3.43)	4 (4.08)	2 (1.75)	2 (9.52)	0.18
Transcatheter aortic valve implantation	1 (0.43)	0 (0)	0 (0)	1 (4.76)	0.0066
History of ablation	29 (12.50)	8 (8.16)	21 (18.58)	0 (0)	0.014
**Type of ablation procedure**					
Atrial fibrillation	13 (9.42)	3 (5.36)	10 (14.08)	0 (0)	0.13
Atrial flutter	5 (3.62)	3 (5.26)	2 (2.86)	0 (0)	0.62
Ventricular tachycardia	6 (4.41)	1 (1.79)	5 (7.25)	0 (0)	0.25
Premature ventricular contractions	6 (4.38)	0 (0)	6 (8.57)	0 (0)	0.050
**Device therapy, n (%)**					
Pacemaker	23 (10.04)	7 (7.37)	16 (14.29)	0 (0)	0.066
ICD	116 (51.10)	30 (31.91)	66 (59.46)	20 (90.91)	<0.001
ICD for primary prevention	86 (76.11)	22 (73.33)	46 (73.02)	18 (90.00)	0.28
Cardiac resynchronization therapy	43 (19.03)	8 (8.51)	26 (23.64)	9 (40.91)	<0.001

* Aortic valve, mitral valve, tricuspid valve.

## Data Availability

The data presented in this study are available on request from the corresponding author. The data are not publicly available due to privacy issues.

## Abbreviations

ACEI	angiotensin-converting-enzyme inhibitor
ARB	angiotensin receptor blocker
ARNI	angiotensin receptor neprilysin inhibitor
BB	beta blocker
BMI	body mass index
CRT	cardiac resynchronization therapy
EF	ejection fraction
EMA	European Medicines Agency
ESC	European Society of Cardiology
FDA	U.S. Food and Drug Administration
HF	heart failure
HFmrEF	heart failure with mildly reduced ejection fraction
HFrEF	heart failure with reduced ejection fraction
HR	heart rate
ICD	implantable cardioverter defibrillator
I_F_	funny channel
LVAD	left ventricular assist device
LVEF	left ventricular ejection fraction
MRA	mineralocorticoid receptor antagonist
NT-proBNP	N-terminal pro-brain natriuretic peptide
NYHA	New York Heart Association
SGLT2	sodium glucose linked transporter 2
SR	sinus rhythm
